# *P. aeruginosa* colonization at ICU admission as a risk factor for developing *P. aeruginosa* ICU pneumonia

**DOI:** 10.1186/s13756-017-0197-9

**Published:** 2017-04-20

**Authors:** Fleur P. Paling, Martin Wolkewitz, Pieter Depuydt, Liesbet de Bus, Frangiscos Sifakis, Marc J. M. Bonten, Jan A. J. W. Kluytmans

**Affiliations:** 1 Julius Center for Health Sciences and Primary Care, University Medical Center Utrecht, P.O. Box 85500, Utrecht, 3508 GA The Netherlands; 2 Institute for Medical Biometry and Statistics, University Medical Center Freiburg, Freiburg, Germany; 3 Department of Intensive Care Medicine, University Hospital of Ghent, Ghent, Belgium; 4 AstraZeneca LP, Gaithersburg, MD USA; 5 Department of Medical Microbiology, University Medical Center Utrecht, Utrecht, The Netherlands

## Abstract

**Objective:**

To determine the incidence of *P. aeruginosa* (PA) ICU pneumonia and its independent association with PA colonization at ICU admission.

**Methods:**

This was a post-hoc analysis of a prospectively collected cohort study. Adult ICU patients with a length of stay of ≥48 h were included and assessed for microbiologically confirmed PA ICU pneumonia. Multivariate survival analysis was performed, including the covariates age, gender, PA colonization at ICU admission, ICU admission specialty and mechanical ventilation at ICU admission, while taking into account the effect of competing risks.

**Results:**

We included 5093 patients, 2447 (48%) were tested for colonization; of those 226 (9.2%) were PA colonized at ICU admission. The incidence of PA ICU pneumonia was 1.34% (*n* = 68). PA colonization was an independent risk factor (subdistribution hazard ratio [SHR] 8.8; 95% confidence interval [CI] 4.9–15.7), as was mechanical ventilation (SHR 5.3, 95% CI 2.7–10.6).

**Conclusion:**

In this study the incidence of *P. aeruginosa* ICU pneumonia was 1.34%. Hazard ratios for PA colonized patients compared to non-colonized to develop PA ICU pneumonia were 8.8. The high risk associated with *P. aeruginosa* colonization for subsequent infection may offer a target for future interventions.

## Introduction

*P. aeruginosa* (PA) is a frequently occurring nosocomial pathogen, causing potentially life threating infections, one of them being Intensive Care Unit (ICU) pneumonia, or pneumonia acquired while hospitalized on the ICU [1, 2]. PA colonization might be a risk factor for PA ICU pneumonia, but the bacterium may also be an innocent bystander in patients with pneumonia caused by another pathogen [1, 3, 4]. The association between PA carriage on ICU admission and the occurrence of PA ICU pneumonia remains relatively unexplored.

## Objective

To estimate the incidence of PA ICU pneumonia and its independent association with PA colonization at ICU admission.

## Methods

This analysis was performed on the data of a prospectively collected observational cohort study, performed in a mixed ICU of a tertiary hospital in Belgium. Data on epidemiology of ICU-acquired infections were collected from January 2010 until June 2014, by means of the locally developed COSARA software application, allowing a continuous prospective registration of all infection- and antibiotic-related data [5].

Adult patients with a length of stay of ≥48 h were included; screening for PA was part of routine care in patients with an expected length of stay of ≥48 h and was based on endotracheal aspirate (ETA), oropharyngeal and/or rectal cultures. Pneumonia diagnosis was based on radiologic criteria in combination with at least 1 clinical or laboratory criterion. Confirmed PA ICU pneumonia cases are those with pneumonia occurring ≥48 h after ICU admission *and* laboratory isolation of PA from any location in the lower respiratory tract. All PA pneumonia diagnoses were cross-validated by trained research physicians. More information on the methods of this analysis are described elsewhere [6].

Patients were regarded as PA colonized at ICU admission if there was a PA positive screening sample or in case of another PA positive respiratory/skin sample on ICU admission ±2 days *and* if there was no PA infection diagnosed on these days. The incidence density of PA ICU pneumonia was determined using a Cox survival analysis that allows controlling for competing events for the occurrence of ICU pneumonia, in this case ICU discharge/death without ICU pneumonia. The final model yielded subdistribution hazard ratios (SHRs) reflecting the relative effect estimates that account for competing events and the other covariates included in the model. The included covariates were age (as a continuous variable), gender, PA colonization at ICU admission, ICU admission specialty (medical vs. surgical) and mechanical ventilation at ICU admission.

## Results

**Fig. 1:**
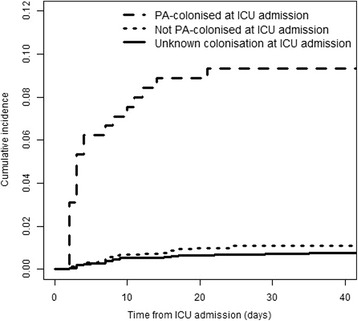
Cumulative incidence function. Cumulative risk of acquiring *P. aeruginosa* ICU pneumonia

Data were collected from 5093 patients, of whom baseline characteristics can be found in Table 1. A total of 2447 patients (48%) were tested for PA colonization at ICU admission; of those 226 (9.2%) were PA colonized. A total of 675 (13.3) patients developed ICU pneumonia. Microbiologically confirmed PA ICU pneumonia occurred in 68 patients (1.34%). In PA colonized patients PA ICU pneumonia occurred in 9.3% (*n* = 21); in confirmed non-colonized it occurred in 1.1% (*n* = 25). The median time to PA ICU pneumonia was 7 days. PA colonization was a risk factor for the development of PA ICU pneumonia with a cause-specific hazard ratio (CSHR) of 9.6 (95% CI 5.3–17.2, *p* < 0.001). Mechanical ventilation at ICU admission was associated with higher CSHR for developing PA ICU pneumonia (CSHR 2.9; 95% CI 1.4–6.0; *p* = 0.004). After accounting for competing events, PA colonization at admission remained a risk factor for the development of PA ICU-pneumonia (SHR 8.8, 95% CI 5.0–15.7, *p* < 0.001, Table 1, Fig. 1), as was mechanical ventilation at ICU admission (SHR 5.3, 95% CI 2.7–10.5, *p* < 0.001).

**Table 1:** Baseline characteristics and subdistribution hazard ratio (SHR) for *P. aeruginosa* ICU pneumonia

	*N* (%) *or mean* (*SD*)	*SHR* (*95* % *CI*)	*p*
Gender: female^a^	1915 (37.6)	0.73 (0.43–1.24)	0.24
Age^b^	59.4 (16.1)	0.99 (0.98–1.01)	0.43
Length of stay in days	9.1 (11.7)	-	-
Median	5.0		
Medical admission^c^	2176 (42.7)	1.34 (0.82–2.20)	0.24
Colonization status at ICU admission			
- PA –	2221 (43.6)	ref	ref
- PA +	226 (4.4)	8.84 (4.96–15.74)	**<0.001** ^*^
- Unknown/missing	2645 (51.9)	1.04 (0.58–1.86)	0.89
ICU mortality	691 (13.6)	-	-
Mechanical ventilation at ICU admission	2591 (50.9)	5.2 (2.70–10.47)	**<0.001** ^*^
ICU pneumonia	675 (13.3)	-	-
- PA	68 (1.3)		
- other confirmed pathogen	347 (6.8)		
- unknown pathogen	260 (5.1)		

^a^male gender is reference category, ^b^SHR per extra year of age, ^c^surgical admission is reference category

^*^significant at the 0.05 level

- = not measured/not applicable

## Discussion

In this study PA colonized ICU patients with a length of stay of ≥48 h had an almost nine times higher risk of developing PA ICU pneumonia than non-colonized patients. Studies that investigate PA colonization as a risk factor for subsequent PA infection are very scarce, and they do not perform multivariate time-to-event analysis in combination with competing risk analyses [3, 4, 7–9].

This study has several limitations, one of them being a single-center study, another being the fact that only half of the patients were tested for PA colonization at ICU admission. Reasons for not testing included the anticipated short stay on ICU for post-surgical patients. Unfortunately, reasons for admission were not recorded and thus we cannot validate this explanation. A second draw-back is the relatively high number of pneumonias caused by unknown pathogens. We cannot rule out that these were caused by (non-cultured) *P. aeruginosa*. However, we performed a sensitivity analyses taking into account pneumonias caused by other and unknown pathogens as competing events, to assess if this changed our final estimates. This was not the case.

Despite the limitations, this study suggests that previous PA colonization contributes to the development of PA ICU pneumonia. Identifying patients at higher risk for developing subsequent infection is important in case that preventive medication becomes available, but also when empirical therapy needs to be started.

## Conclusion

In this study the incidence of *P. aeruginosa* ICU pneumonia was 1.34%. Hazard ratios for PA colonized patients compared to non-colonized to develop PA ICU pneumonia were 8.8.
